# Improvement of mechanical properties of HfB_2_-based composites by incorporating in situ SiC reinforcement through pressureless sintering

**DOI:** 10.1038/s41598-021-88566-0

**Published:** 2021-05-10

**Authors:** S. Ghadami, E. Taheri-Nassaj, H. R. Baharvandi, F. Ghadami

**Affiliations:** 1 Department of Materials Science and Engineering, Tarbiat Modares University, PO Box 14115-143, Tehran, Iran; 2 School of Metallurgy and Materials, College of Engineering, University of Tehran, Tehran, Iran

**Keywords:** Materials science, Chemical synthesis

## Abstract

HfB_2_, Si, and activated carbon powders were selected to fabricate 0–30 vol% SiC reinforced HfB_2_-based composite. Pressureless sintering process was performed at 2050 °C for 4 h under a vacuum atmosphere. Microstructural studies revealed that in situ SiC reinforcement was formed and distributed in the composite according to the following reaction: Si + C = SiC. A maximum relative density of 98% was measured for the 20 vol% SiC containing HfB_2_ composite. Mechanical investigations showed that the hardness and the fracture toughness of these composites were increased and reached up to 21.2 GPa for HfB_2_-30 vol% SiC and 4.9 MPa.m^1/2^ for HfB_2_-20 vol% SiC, respectively. Results showed that alpha-SiC reinforcements were created jagged, irregular, and elongated in shape which were in situ formed between HfB_2_ grains and filled the porosities. Formation of alpha-SiC contributed to improving the relative density and mechanical properties of the composite samples. By increasing SiC content, an enhanced trend of thermal conductivity was observed as well as a reduced trend for electrical conductivity.

## Introduction

Advanced materials and coatings which are operating in a high-temperature environment have been an attractive issue in the materials research area^1–19^. With a melting point > 3000 °C, ultra-high temperature ceramics (UHTCs) belong to structural advanced ceramics which have great potential for high-temperature applications^20–22^.

Among UHTCs, HfB_2_ ceramic include both metal-like and ceramic-like properties. High thermal conductivity, moderate coefficient of thermal expansion (CTE), high elastic modulus, and hardness refer to ceramic-like properties of HfB_2_^23^. On the other side, high electrical conductivity refers to the metal-like of HfB_2_ ceramic. A combination of excellent thermophysical properties, mechanical properties, and oxidation resistance of HfB_2_ are superior to other di-borides such as ZrB_2_ and TiB_2_. Advanced sintering methods such as spark plasma sintering, microwave sintering, and hot pressing have been developed to achieve a fully dense HfB_2_-based composite. Even though, these methods could not be suitable for complex geometries of large-size specimens. Pressureless sintering could be an appropriate method for this purpose.

By incorporating SiC as reinforcement to HfB_2_, mechanical properties of the monolithic HfB_2_ are enhanced^24,25^. It has been reported that SiC second phase has a beneficial effect on the mechanical properties of HfB_2_ ceramic^26^. Wang et al.^27^ fabricated HfB_2_–SiC composite using HfSi_2_, B_4_C, and carbon as starting materials where both matrix and reinforcement were formed as the following reaction:

1 $${\text{2HfSi}}_{{2}} + {\text{ B}}_{{4}} {\text{C }} + {\text{ 3C }} = {\text{ 2HfB}}_{{2}} + {\text{ 4SiC}}$$

They demonstrated and reported the Vickers hardness of 20.4 GPa and the fracture toughness of 4.7 MPa.m^1/2^ for HfB_2_–SiC composite. Monteverde^24^ prepared HfB_2_–SiC composite by the reactive hot-press method. He showed that HfB_2_ and SiC phases could be formed according to the following reaction:

2 $${\text{Hf }} + {\text{ Si }} + {\text{ B}}_{{4}} {\text{C }} = {\text{ HfB}}_{{2}} + {\text{ SiC }} + {\text{ HfC}}$$

In a similar study Lee et al. ^28^ produced HfB_2_–SiC composite according to reaction (3) as following:

3 $${\text{Hf }} + {\text{ Si }} + {\text{ B}}_{{4}} {\text{C }} = {\text{ HfB}}_{{2}} + {\text{ SiC}}$$

They reached the maximum relative density of 99.8%, the fracture toughness of 5.3 MPa.m^1/2^_,_ and the Vickers hardness of 18.3 GPa using a reactive hot pressing method at 1900 °C for the composite. Table 1 represents a comparative study on the properties of the SiC reinforced HfB_2_-based composites with respect to the material compositions.

**Table 1:** Preparation conditions and properties of SiC reinforced HfB_2_-based composites.

Material composition	Starting powders	Sintering condition	Microstructural phases	Relative density (%)	Hardness (GPa)	Fracture toughness (MPa.m^1/2^)	Grain size (μm)	Ref
HfB_2_-30 vol% SiC	HfB_2_, SiC	SPS, 2100 °C, 30 MPa, 2 min,	HfB_2_, SiC, HfO_2_	100	26	3.9	2	^29^
HfB_2_-20 vol% SiC	HfB_2_, SiC, B_4_C, C	HP, 2000 °C, 20 MPa, 45 min	HfB_2_, SiC	98.2	–	–	–	^30^
HfB_2_-20vol.% SiC-5vol.% WC	HfB_2_, SiC, B_4_C, C, WC	HP, 2000 °C, 30 MPa, 1 h	HfB_2_, SiC, Hf,W)B_2_, (Hf,W)C	99	22.3	3.76	1.5	^31^
HfB_2_-10vol.% SiC- 10vol.% HfC	HfB_2_, SiC, HfC	HP, 2000 °C, 30 MPa, 1 h	HfB_2_, SiC, HfC	98.8	20.17	3.36	2.97	^32^
HfB_2_-SiC-MoSi_2_	HfB_2_, Si, Mo, C	RSPS, 1850 °C, 5 min, 40 MPa	HfB_2_, SiC, MoSi_2_, HfC	98.6	25.2	5.1	3.25	^1^
HfB_2_–SiC–VSi_2_	HfB_2_, VC, Si	PS, 2150 °C, 4 h	HfB_2_, SiC, VSi_2_, HfC	98	20.1	5.8	10	^33^
HfB_2_-TiB_2_-SiC-MoSi_2_	HfB_2_, TiB_2_, Si, Mo, C	PS, 2050 °C, 5 h	(Hf,Ti,Mo)-B, (Hf,Ti,Mo)-C, SiC, MoSi_2_, HfC	98	23.2	5.4	–	^34^
HfB_2_-SiC	HfSi_2_, B_4_C, C	RSPS, 1600 °C, 40 MPa, 10 min	HfB_2_, SiC,	98.7	20.4	4.7	2	^27^
HfB_2_-30 vol% SiC	HfB_2_, SiC	RSPS, 1800 °C, 40 MPa, 15 min	HfB_2_, SiC	98.9	15.31	5.75	5.3	^35^
HfB_2_-SiC-15 vol%HfC	HfO_2_, Mg, H_3_BO_3_, C, Si	SPS, 1850 °C, 30 MPa, 5 min	HfB_2_, SiC, HfC	99.97	22.7	4.98	–	^36^

The majority number of studies on the HfB_2_-SiC has been focused on the formation matrix and reinforcement simultaneously and evaluating its mechanical properties. In this work, for the first time, in situ HfB_2_–SiC composites are fabricated by pressureless sintering at 2050 °C for 4 h, where despite other researches, only SiC reinforcement is formed during the sintering process. We discuss the effect of in situ SiC content on the physical and mechanical properties of HfB_2_–SiC composites.

## Experimental methods

Commercial HfB_2_, Si, and activated carbon as the starting powders were selected to synthesis HfB_2_-based composites. The characteristics of starting powders are listed in Table 2. In order to achieve the final composition, the volume fractions were calculated according to the theoretical density of 11.2 g/cm^3^ for HfB_2_ and 3.2 g/cm^3^ for SiC. Specification and the sintering conditions of the composite samples are shown in Table 3.

**Table 2:** Specification of the starting powders before sintering.

Row material	Purity (%)	Mean particle size (µm)	Impurities	Vendor
HfB_2_	95	20	HfO_2_	Beijing Cerametek Materials (China)
Si	99.5	10	–	Merck (Germany)
Activated Carbon	99	10	–	Shanghai activated carbon (China)

**Table 3:** Characterizations and sintering conditions of in situ HfB_2_-SiC composites sintered at 2050 °C for 4 h.

Sample code	SiC (vol%)	wt% of starting powders			Density		
		HfB_2_	Si	C	Theoretical density (g/cm^3^)	Bulk density (g/cm^3^)	Relative density (%)
HS0	0	100	0	0	11.2	10.25	91.5
HS5	5	98.51	1.03	0.44	10.8	10.16	94.1
HS10	10	96.92	2.15	0.92	10.4	9.9	95.2
HS15	15	95.2	3.36	1.44	10	9.66	96.6
HS20	20	93.33	4.66	2	9.6	9.41	98
HS25	25	91.3	6.08	2.6	9.2	8.99	97.7
HS30	30	89.09	7.63	3.27	8.8	8.55	97.1

At the initial milling step, Si and C powders were milled by a high-energy planetary mill for 5 h in ethanol medium. WC–Co milling media were used and a speed ratio of the milling process was defined 300 rpm. The weight ratio of powders to balls was determined 1:3.

To remove ethanol from mixed powders, the dry process of mixing powders was accomplished for 24 h in the air. In the next milling step, the mixed powders (Si + C) were added to HfB_2_ powder and then were milled for another 3 h at the above conditions. The final slurry of mixed powders (HfB_2_ + Si + C) was dried for 24 h in the air.

The cylindrical samples (Φ25 × 8 mm^2^) without any binders were cold-pressed by uniaxial pressing at 50 MPa and then were cold isostatically pressed at 300 MPa. Reactive pressureless sintering process was performed in a commercial graphite resistance heating furnace at 2050 °C for 4 h under a vacuum atmosphere of 0.05 mbar.

In the next stage, the sintered samples were grounded using a cubic born nitride (CBN) rotating disk and then were polished by SiC abrasive papers and a fine diamond paste until the surface of samples was mirror‐like. The bulk density of sintered samples was measured using Archimedes method. Hence, the relative density of the sintered samples was reported by the ratio between the bulk and theoretical density.

Phase analysis was carried out using X-ray diffraction pattern (XRD, Philips, Model: X'Pert MPD, Tube: Co, and λ: 1.78897 Å). The microstructure of the sintered samples was investigated by a field emission scanning electron microscope (FESEM, TESCAN, Model: MIRA3) equipped with energy-dispersive spectroscopy (EDS). It should be noted that the microstructure of samples was directly examined without the thermal or chemical etching step.

Vickers hardness was measured by a Vickers indenter with 0.5 kg applied load on polished sections, according to ISO6507 standard. To ensure results' reliability, 20 indentations were made and 40 diagonal lengths were measured for each specimen. The fracture toughness ($KIC$) calculations were done using Evans and Charles equation based on the measurements of the radial crack length produced by Vickers^37^:

4 $$K{\text{IC}}=0.16 {(c/a)}^{-3/2}{ (Ha}^{1/2})$$

where $KIC$ is the fracture toughness (MPa.m^1/2^), H means Vickers hardness (GPa), *c* is the average half-length of the crack acquired in the tips of the Vickers marks (m), and *a* is the average half-length of indentation diagonal (m). The grain size of the samples was determined based on the line intercept method (ASTM E112-13) utilizing ImageJ software. Young's modulus was determined through ultrasonic testing at 25 °C according to the ASTM C1198 by sound velocity using the TC600 model thickness measuring apparatus. The electrical conductivity of the samples was measured by commercial equipment according to the standard four-point probe method at ambient temperature to reduce the effects of contact resistance. The thermal conductivity of the samples was determined according to the following equation proposed by Parker et al.^38^:

5 $${\text{km}} = \alpha \rho {\text{Cp}}$$

where km is thermal conductivity, α is the thermal diffusivity, ρ is the density, and Cp is the heat capacity. The thermal diffusivity was measured by the laser flash method for cylindrical-shaped samples at room temperature.

## Result and discussion

### Characterization and preparation conditions of materials

**Figure 1:**
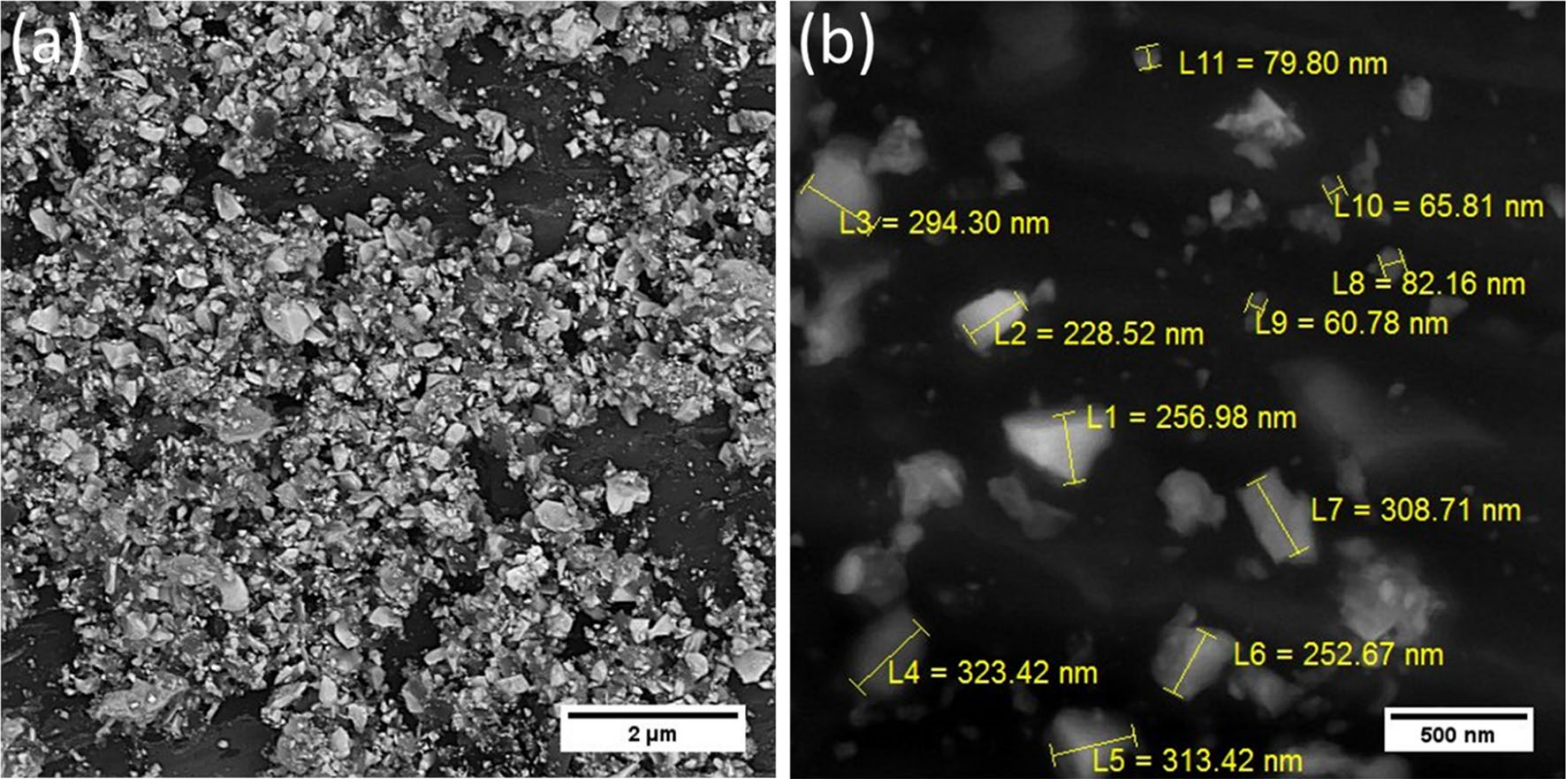
FESEM micrographs of mixed powders after 8 h milling process (**a**) low magnification and (**b**) high magnification.

Figure 1 represents the size and morphology of the mixed powders after the milling process. The powders were homogenously mixed by a high-energy planetary mill. Moreover, the size of mixed powders was reduced and reached below 1 μm as well as some particles reached the nano-metric scale according to measurements made by ImageJ software. Sayyadi-Shahraki et al.^3^ reported that the particle size of the powders before sintering has a significant effect on the density and mechanical properties of the composite. It concluded that the milling process had a key role in the densification process and the formation in situ phase during sintering. On the other word, the combination of Si and C powders in the initial milling step as well as dispersing of Si and C powders on the main HfB_2_ powder at the next milling step increased the possibility of the reaction between Si and C according to the following reaction:

6 $${\text{Si }} + {\text{ C }} = {\text{ SiC}}$$

**Figure 2:**
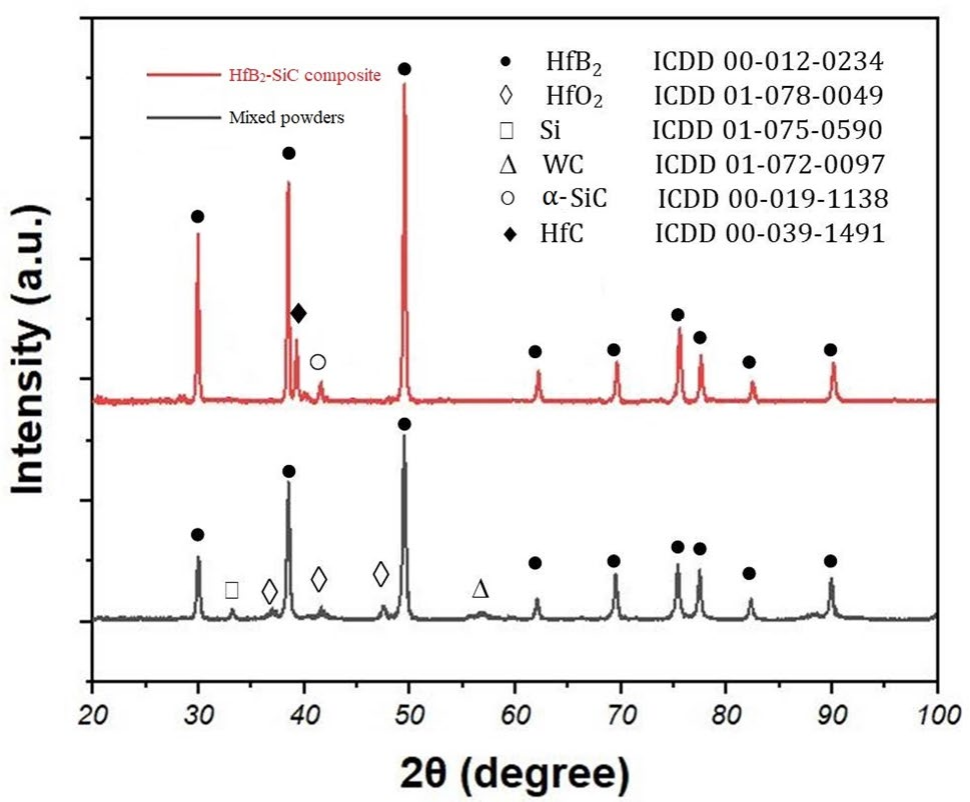
XRD patterns of mixed powders after 8 h milling process and HS20 after the sintering process for 4 h at 2050 °C.

Figure 2 illustrates the detected phases of mixed powders (HfB_2_ + Si + C) by XRD analysis. No combination and reaction between powders were observed after the milling process. The phases from the starting powders were detected as well as HfO_2_ and WC phases. It can be expected that HfO_2_ phase was detected from HfB_2_ impurities. Besides, WC phase could come from WC–Co media during the milling process. These results are agreeing with the findings of other researchers^1,2,39^.

### Microstructural analysis

**Figure 3:**
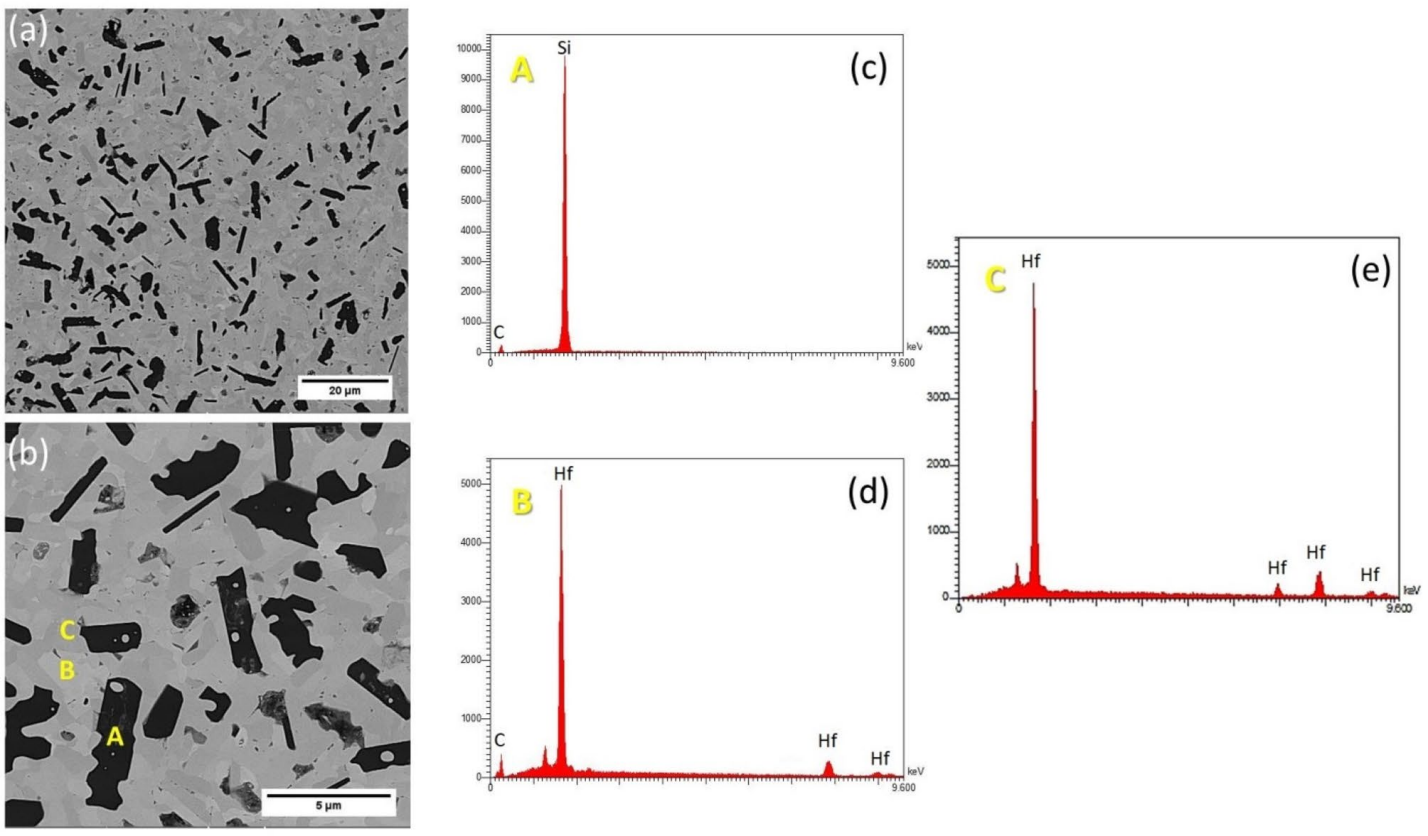
Backscattered image of HS20 (**a**) low magnification, (**b**) high magnification, (**c**) EDS pattern spot scan for A, (**d**) EDS pattern spot scan for B, and (**e**) EDS pattern spot scan for C.

The microstructure of the composite and related EDS analysis are shown in Fig. 3.

In situ SiC reinforcement was successfully formed and distributed in the HfB_2_ skeleton according to EDS analysis. Furthermore, HfC phase could form from the following sub-reactions:

7 $${\text{HfO}}_{{2}} + {\text{3C }} = {\text{HfC}} + {\text{ 2CO}}\left( {\text{g}} \right)$$

8 $${\text{HfO}}_{{2}} + {\text{ 3WC }} = {\text{ HfC}} + {\text{ 3W }} + {\text{2CO}}\left( {\text{g}} \right)$$

In the previous study, the thermodynamic formation of SiC and HfC phases was discussed^1^. Back to the detail of the main reaction (6), the possibility of this reaction was proved in an inert atmosphere^40^. The formation of in situ SiC reinforcement was an effective role in eliminating the porosity of the composite.

As a hypothesis the in situ SiC was formed in hollow spaces between HfB_2_ grains. Based on this hypothesis, by increasing in situ SiC phase, the porosity content was decreased. This result was supported by the density measurements. The detected phases of the sintered composites are shown in Fig. 2. No trace of the oxide and the impurity phases were detected by XRD analysis. On the other hand, Si, C, and WC peaks were not observed in the XRD pattern of the composites after the sintering process. By detecting SiC and HfC phases, it should be concluded that the reactions (6), (7), and (8) were mostly progressed.

**Figure 4:**
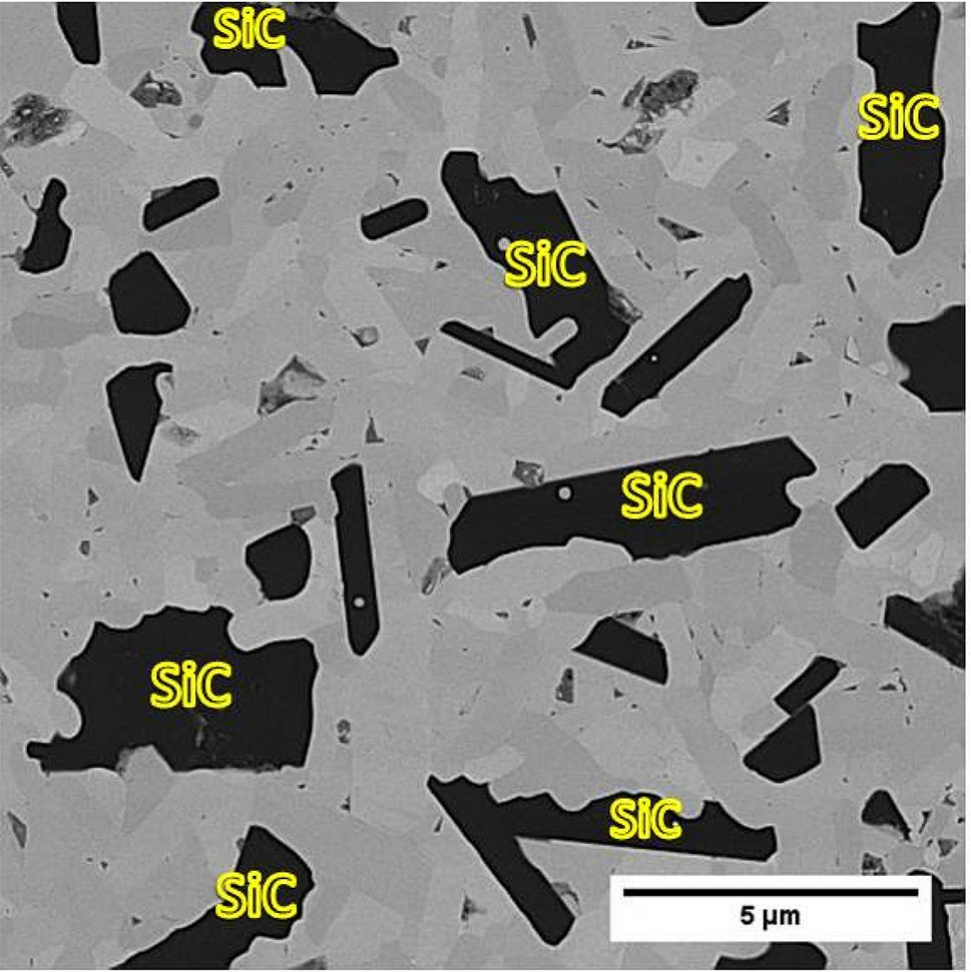
Backscattered micrograph of HS20. The elongated and irregular shape of in situ SiC reinforcement is observed.

The closer view of in situ SiC reinforcements is shown in Fig. 4. In situ SiC phase was created in the form of elongated and irregular shapes. Since the formation reaction of SiC (reaction (6)) is accomplished at high-temperature (> 1650 °C)^34^, it can be expected that in situ SiC phase would be formed in alpha-SiC polymorph. In Fig. 4, it can be clearly seen that some in situ SiC grains are jagged. The jagged, irregular, and elongated shape of in situ SiC proved the following items:

1. Alpha-SiC phase was formed during the sintering process
2. Alpha-SiC grains were in situ formed between HfB_2_ grains and filled the porosities. Hence, the formation of in situ SiC reinforcement contributed to the desired density of the composite samples (see Sect. 3.2).
3. Alpha-SiC reinforcement contributed to enhancing the fracture toughness of the composite by acting as a barrier against crack propagation (see Sect. 3.3).

**Figure 5:**
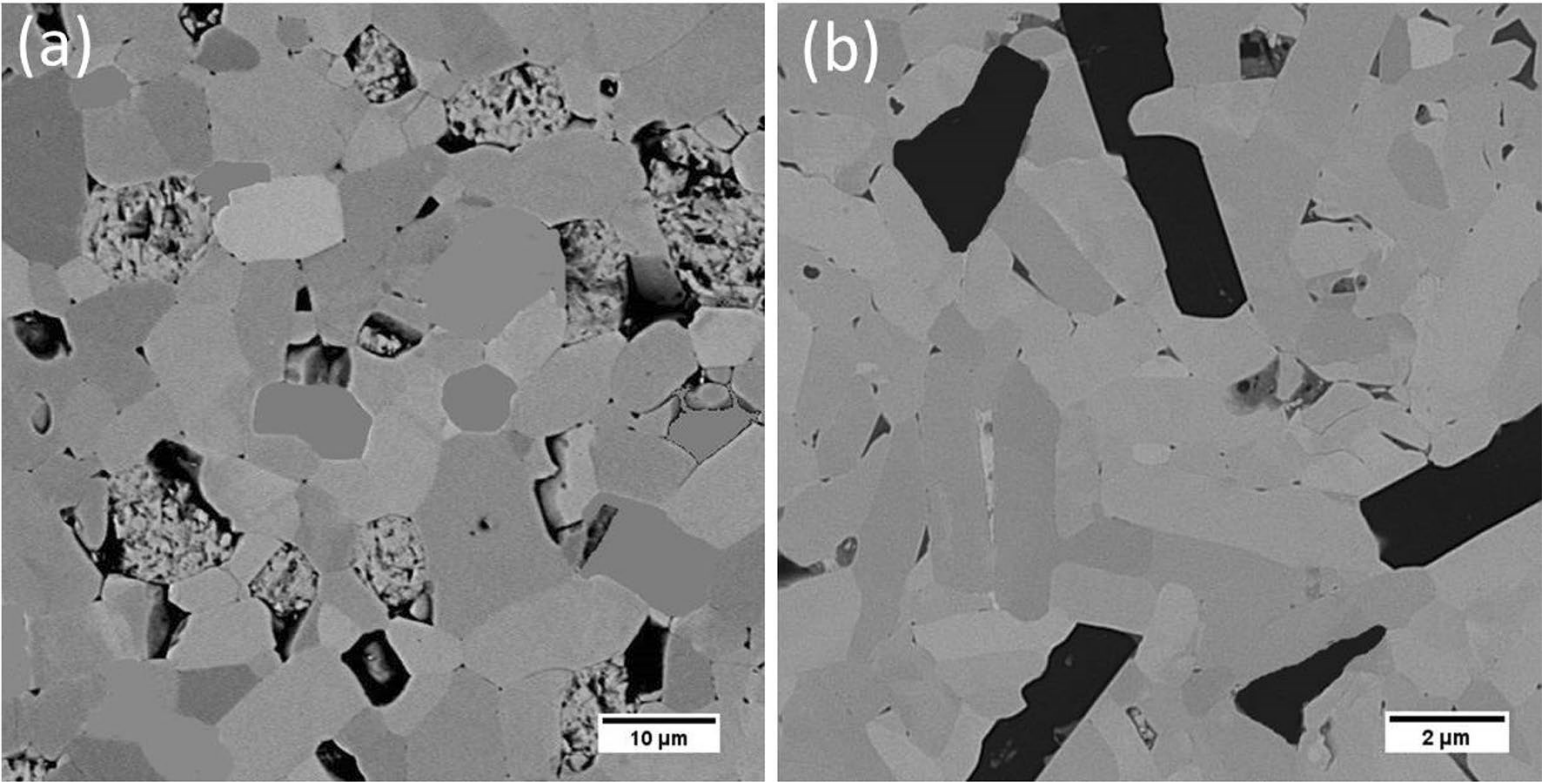
FESEM micrographs of (**a**) Hf0 and (**b**) Hf25. Showing reduction of HfB_2_ grain size by in situ formation of SiC reinforcement.

Figure 5 shows the microstructure of monolithic HfB_2_ (Hf0) and HfB_2_-25 vol% SiC (Hf25). Some porosity, as well as relatively large size of HfB_2_ grain (about 12 μm), can be observed from Fig. 5a. On the other side, dens microstructure and relatively small grain size of HfB_2_ grain (about 5 μm) can be seen from Fig. 5b. This finding supports calculated results from density and grain size measurements.

### Physical properties

**Figure 6:**
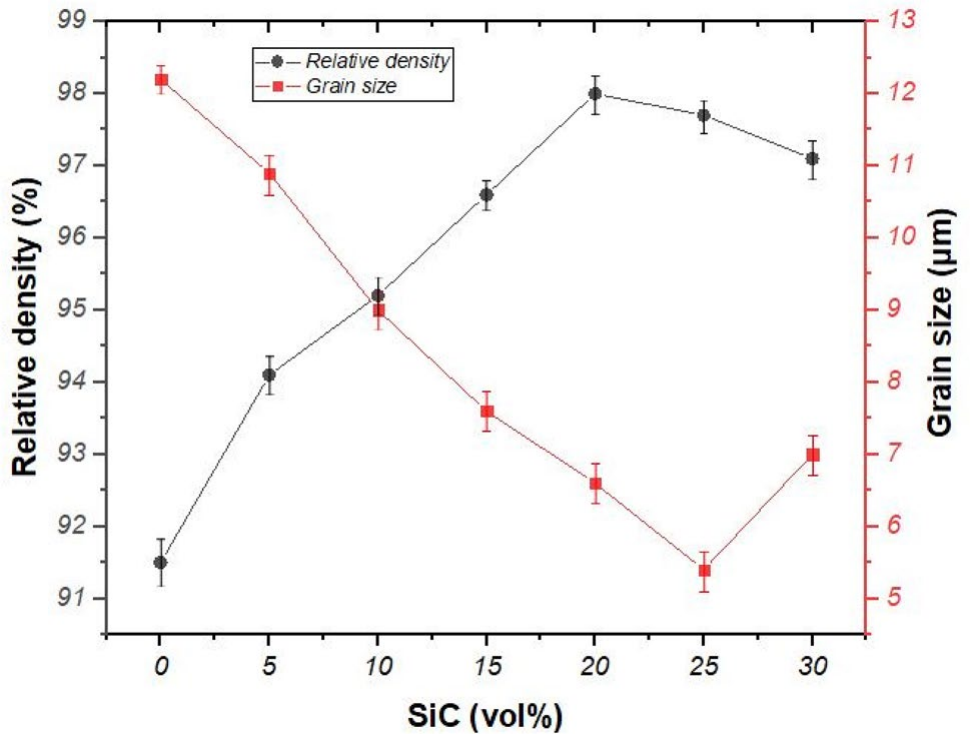
Plots depicting the variation of the relative density and grain size of in situ HfB_2_–SiC composites.

The relative density of the composites is plotted in Fig. 6. The relative density of monolithic HfB_2_ (HS0) was measured 91.5%. The covalent nature of HfB_2_ causes the poor sinterability of this ceramic^41–43^. It seems that the formation of in situ HfC phase increased the relative density of Hf0 compared to other similar researches for monolithic HfB_2_ (~ 80%)^44^. It is noticeable that the relative density increased in the case of volume percentages which are lower than 20% and reached a peak of 98% for HS20. It seems that was because of the borosilicate liquid phase (B_2_O_3_‐SiO_2_) formation which facilitated the sintering mechanism. This borosilicate liquid phase was expected to form during the sintering process (up to 2050 °C) due to the melting points for the impurities of Si (SiO_2_) and HfB_2_ (B_2_O_3_) which are 450 °C and 1710 °C, respectively. Moreover, the proportion of this liquid phase went up by rising the volume percentage of the SiC phase and led to modification on the densification process of the composite. Apart from this enhancement, in situ SiC reinforcement filled the space between the HfB_2_ grains thereby improving the densification process. Figure 6 also shows a decrease in the relative density in the SiC volume percentages which were higher than 20%. Mashhadi et al.^45^ reported that the agglomeration of SiC reinforcement could lead to reducing the relative density of ZrB_2_-SiC. It seems that the SiC content up to 20vol% effectively promoted the densification of HfB_2_–SiC composites and caused it to reach 98%. In addition, the ultra-fine powder from the high-energy milling process also caused to increase the relative density of samples.

The effect of SiC content on the grain size of HfB_2_–SiC composites is shown in Fig. 6. The grain size of HS0 was 12.2 μm which indicated that the raising temperature up to 2050 °C causes the grain growth of HfB_2_ grains. By increasing SiC content, the size of HfB_2_ grains decreased and reached 5.4 μm for Hf25. The migration of grain boundary was restricted due to the formation of in situ SiC reinforcement and ended up with a smaller size of matrix grains. It is worth noting that in situ SiC reinforcement played a role as a barrier against the growth of HfB_2_ grains.

Wang et al.^27^ reported that lower sintering temperature and shorter holding time contribute to the small grain of HfB_2_. It should be concluded that the temperature of 2050 °C and holding time of 4 h was enough to reach the desired relative density as well as the reduced grain size of the HfB_2_-SiC composites.

**Figure 7:**
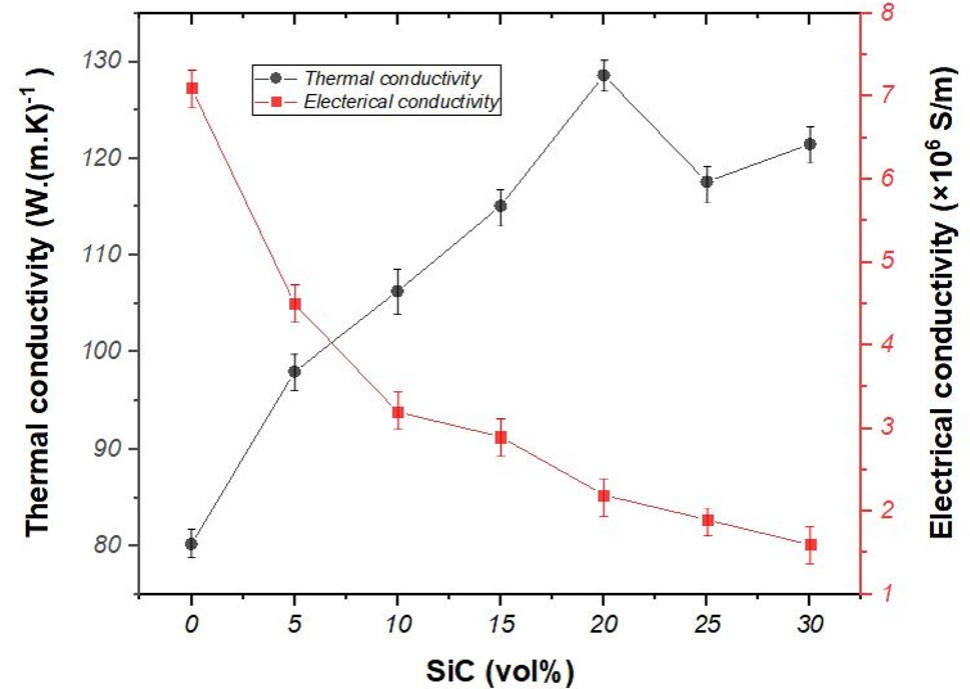
The variation of electrical and thermal conductivity of in situ HfB_2_–SiC composites.

Figure 7 shows the variation of electrical and thermal conductivity of the composite samples. The electrical conductivity of the composite samples decreased with increasing SiC content. The electrical conductivity of HS0 and HS30 was measured about 7.1 × 10^6^ and 1.6 × 10^6^ S/m, respectively.

It is well-known HfB_2_ is conductive and its electrical conductivity has been reported about 9.1 × 10^6^ S/m^46^.

Although the higher density of composite was expected to increase the electrical and thermal conductivity, the electrical conductivity of the composite dwindled as a result of the low electrical conductivity of SiC (about 1.32 × 10^−4^ S/m^47^). In Fig. 7, an enhancing trend of the thermal conductivity is observed by increasing SiC content up to 20vol%. The thermal conductivity of HS0 and HS20 was measured about 80.2 and 128.6 W (m K)^-1^, respectively. It also can be found; using more than 20 vol% of the SiC resulted in decreasing the thermal conductivity of the composite. Culter et al.^23^ reported the thermal conductivity of 104 W (m K)^−1^ for HfB_2_. Moreover, the thermal conductivity of SiC was reported about 490 W (m K)^1^^48^. Based on the rule of mixture, it can be expected the enhancing trend of the thermal conductivity of the composite samples by increasing SiC content. It should be noted that the porosity has a negative effect on thermal conductivity. Hence, the maximum value of the thermal conductivity of the samples refers to the sample with the highest value of the density.

### Mechanical properties

**Figure 8:**
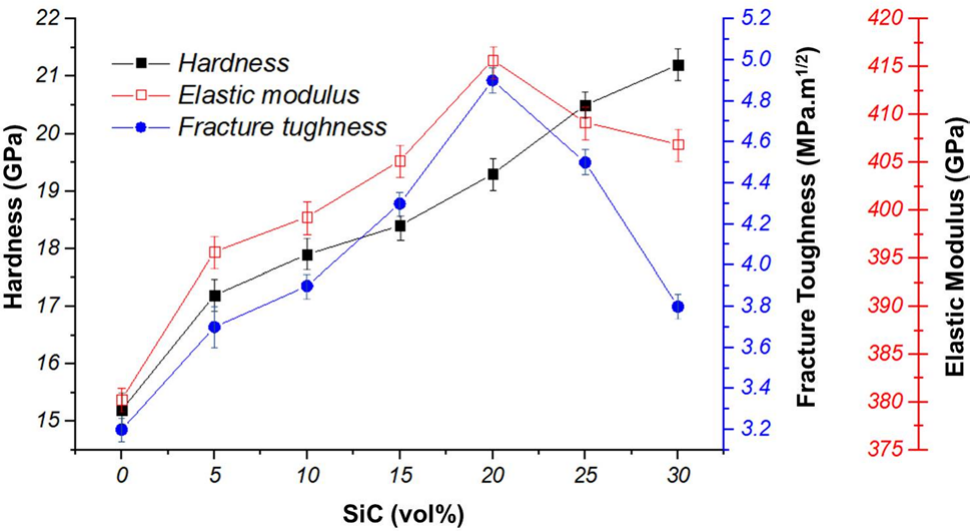
The variation of Vickers hardness, fracture toughness, and elastic modulus of in situ HfB_2_–SiC composites.

The variation of the Vickers hardness of the samples is plotted in Fig. 8. An improving trend of the hardness is observed with increasing vol% of SiC. The hardness value of SiC was reported about 32 GPa which is more than those reported for HfB_2_ (about 21.2Gpa)^23,49^. Therefore, it can be expected that the hardness value of the samples was enhanced by incorporating SiC as reinforcement. Based on the rule of mixture, the hardness value was improved by increasing in situ SiC content. The maximum Vickers hardness was measured about 21.2 GPa for HS30. It is worthy to note that these results were achieved by the pressureless sintering method, whereas some researchers reported the hardness values which were more than 20 GPa using the pressure assistant methods.

Desired density, as well as the good distribution of in situ SiC phase, had an effective role in Vickers hardness of the composite samples. The variation of the fracture toughness of the samples is also plotted in Fig. 8. As can be seen, the fracture toughness of the samples is improved up to 20 vol% of SiC and then decreased by increasing in situ SiC content more than 20vol%. The fracture toughness of the HS20 was measured about 4.9 MPa.m^1/2^. It also reported the shape of the second phase would affect the fracture toughness of the composite^1,3^. Aside from the shape of the second phase, in situ formed phase has a positive effect on the fracture toughness of the composite. Fortunately, in situ SiC reinforcement was formed as alpha polymorph which had needle-like morphology. The synergistic effect of the in situ formation of SiC reinforcement, as well as the elongated shape of the second phase (in situ SiC) could improve the fracture toughness of the composite samples. Balak et al.^50^ reported the fracture toughness of 4.6 MPa.m^1/2^ for ZrB_2_-SiC composites fabricated by spark plasma sintering. They demonstrated that the addition of SiC up to 20 vol% enhances the fracture toughness of the composite. In a similar study, Ghadami et al.^51^ investigated the reinforced composite by SiC. They discovered the fracture toughness value diminished due to using the reinforcement more than a specific proportion. The quality of the interface had also a considerable impact on the fracture toughness value as a weak interface between matrix and the reinforcement would lead to propagating the crack through this way. In fact, the crack tends to propagate through the weak interface, therefore, less energy will waste from the crack, and eventually, the failure will occur. In this study, however, the SiC reinforcement was strongly bonded HfB_2_ matrix. As a result, neither impurity nor the other phases could have formed on the interface. Wang et al.^27^. Mentioned a clean interface and intimated contact between matrix and reinforcement grains were the main characteristic of in situ composite.

The desired fracture toughness of the composite samples is attributed to the energy-wasting of the crack by pinning, deflection, bridging, and branching mechanisms as well as the strong interface between in situ SiC and HfB_2_. The detail on the effect of fracture toughness increasing by incorporating in situ SiC reinforcement to HfB_2_-based composite was reported elsewhere^33^.

The variation of elastic modulus of the composite sample is plotted in Fig. 8. The maximum elastic modulus of the composite samples was calculated about 415.7 GPa for HS20. Based on the rule of mixture, it can be expected that the elastic modulus is improved with increasing in situ SiC content. Besides, the density of the composite has an impact on the elastic modulus in a way in which the porosity of the composite will has a negative effect on the elastic modulus of the composite. Consequently, a fully dense composite contributed to achieving a higher value of elastic modulus. According to the density measurement of the composite samples, maximum relative density was measured for HS20 which can expect that the maximum elastic modulus belongs to the composite sample containing 20 vol% of SiC. Ni et al.^52^ reported the elastic modulus of 489.6 GPa as well as the relative density of 98.6% for HfB_2_-20 vol% SiC which is in close with the findings of this study (elastic modulus of 415 GPa and relative density of 98% for HS20).

**Figure 9:**
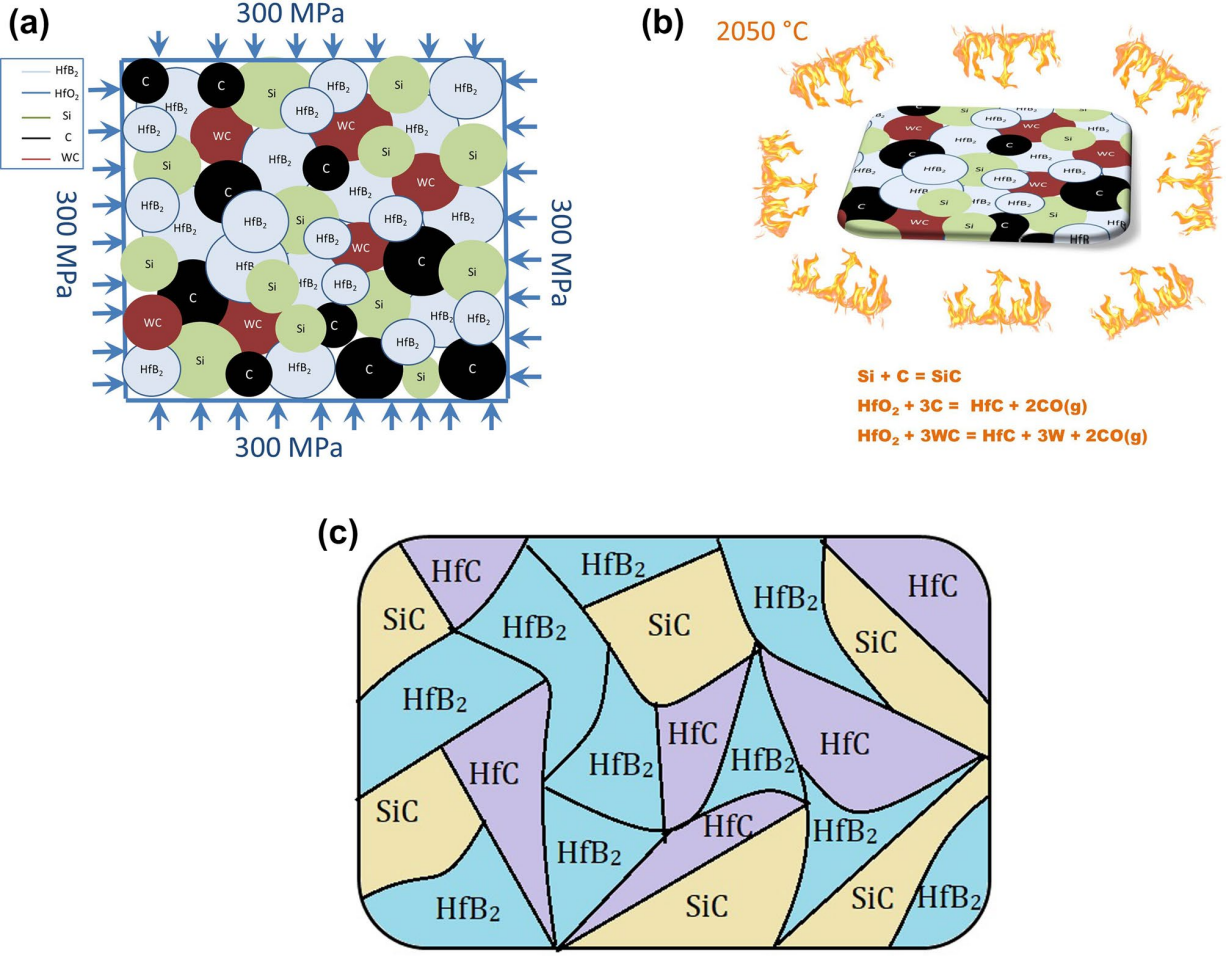
Schematic drawing of the sintering mechanism during reactive consolidation of HfB_2_–SiC composite (**a**) pressing of mixed powders by a cold isostatic press under 300 MPa, (**b**) reaction taking place during sintering process at 2050 °C, and (**c**) final microstructure of the HfB_2_-SiC composite after sintering at 2050 °C including HfB_2_, SiC, and HfC phases.

The sintering mechanism of HfB_2_-SiC composite during the sintering process is schematically illustrated in Fig. 9. After the milling process, mixed powders including HfB_2_ (containing HfO_2_ impurity), Si, and C were randomly distributed. Form Fig. 9a, powder mixtures were cold isostatically pressed under 300 MPa. During the sintering process, the reaction between Si and C could happen and SiC was in situ formed according to reaction (6). Similarly, the reaction between HfO_2_ and C as well as HfO_2_ and WC could progress and HfC was in situ formed according to reaction (7 and 8) (Fig. 9b). Finally, the microstructure of the composite was consist of HfB_2_, SiC, and HfC phases which homogeneously distributed in the microstructure after the sintering process (Fig. 9c).

## Conclusion

In situ HfB_2_-SiC composites were fabricated by pressureless sintering method at 2050 °C for 4 h under a vacuum atmosphere of 0.05 mbar. SiC reinforcement in situ formed and homogeneously distributed in HfB_2_ skeleton after the sintering process. According to results, SiC reinforcements were jagged, irregular, and elongated in shape which proved that alpha-SiC was in situ created during sintering. Based on the morphology of alpha-SiC, enhanced relative density and fracture toughness of the composite samples attributed to the formation of in situ alpha-SiC. The increasing trend of the relative density, thermal conductivity, elastic modulus, and fracture toughness was observed by incorporating in situ SiC content up to 20vol%. The maximum value of Vickers hardness was measured about 21.2 GPa for HS30. In addition, the results that were related to the electrical conductivity demonstrated the negative effect of in situ SiC phase on this parameter due to the dielectric nature of SiC. The grain size of the composite samples was continuously reduced up to 25 vol% of SiC. The minimum grain size of the samples was measured about 5.4 μm for HS25. Indeed, the major cause of improving the mechanical properties of the composites was in situ formation of SiC reinforcement which resulted in the clean interface and intimated contact with HfB_2_ matrix. As a consequence, the findings indicated the huge potential of the modified HfB_2_-SiC composites as a new generation of ultra-high temperature parts and equipment in the future. Despite this, there are still several issues that need to be solved such as detailed microstructural investigation and phase analyses in the interfaces to fulfill their wide application.
